# Genome-Wide Identification and Analysis of the WNK Kinase Gene Family in Upland Cotton

**DOI:** 10.3390/plants12234036

**Published:** 2023-11-30

**Authors:** Qi Zhang, Caidie Zhang, Zhenyuan Pan, Hairong Lin, Zhibo Li, Xinhe Hou, Jinshan Liu, Xinhui Nie, Yuanlong Wu

**Affiliations:** 1 Key Laboratory of Oasis Ecology Agriculture, Xinjiang Production and Construction Crops, Agricultural College, Shihezi University, Shihezi 832003, China; zq10272023@126.com (Q.Z.); 18299243164@163.com (C.Z.); panzhenyuandawood@163.com (Z.P.); linhairongyou@126.com (H.L.); lzb_oea@shzu.edu.cn (Z.L.); 2 Xinjiang Production and Construction Corps Seed Management Station, Urumqi 830011, China; xjhouxinhe@163.com

**Keywords:** WNK family protein, upland cotton, genome-wide, gene expression, biotic stress, abiotic stress

## Abstract

With-No-Lysine (WNK) kinases are a subfamily of serine/threonine protein kinases. WNKs are involved in plant abiotic stress response and circadian rhythms. However, members of the WNK subfamily and their responses to abiotic and biotic stresses in *Gossypium hirsutum* have not been reported. In this study, 26 *GhWNKs* were identified in *G. hirsutum*. The gene structure, conserved motifs, and upstream open reading frames (uORFs) of *GhWNKs* were identified. Moreover, *GhWNKs* regulation is predicted to be regulated by *cis*-acting elements, such as ABA responsive element (ABRE), MBS, and MYC. Furthermore, transcription factors including MIKC_MADS, C2H2, TALE, bZIP, Dof, MYB, bHLH, and HD-ZIP are projected to play a regulatory role in *GhWNKs*. The expression patterns of *GhWNKs* under normal conditions and biotic and abiotic stresses were evaluated, and their expression was found to vary. The expression patterns of several *GhWNKs* were induced by infiltration with *Verticillium dahliae*, suggesting that several *GhWNKs* may play important roles in the response of cotton to *V. dahliae*. Interestingly, a homoeologous expression bias within the *GhWNKs* was uncovered in upland cotton. Homoeologous expression bias within *GhWNKs* provides a framework to assist researchers and breeders in developing strategies to improve cotton traits by manipulating individual or multiple homeologs.

## 1. Introduction

Protein kinases, as the largest superfamily of phosphorylate proteins, play important roles in various cellular processes and physiological functions [1]. Within this superfamily, With-No-Lysine (WNK) kinases represent a subfamily of the serine/threonine protein kinases. They retain intact kinase activity because of the catalytic lysine in kinase subdomain-1 but lack a critical catalytic lysine (K) residue within subdomain-2 [2]. WNK homologs function in various eukaryotes, including animals and plants [2].

There are 11 WNK members in *Arabidopsis thaliana* and 9 WNK members in *Oryza sativa* [3,4]. Cotton, which belongs to the Malvaceae family and *Gossypium* genus, is an important economic crop and is considered a model species for studying polyploidy in plants [5,6]. However, the number of WNKs in upland cotton has not been reported. Many studies have shown that WNK proteins play important roles in plant growth and development. The regulation of circadian rhythms by WNKs has been well characterized, particularly in *Arabidopsis* and *O. sativa* [7,8]. In *Arabidopsis*, WNK1 contributes to circadian rhythms by phosphorylating APRR3, and part of the APRR1/TOC1 quintet is involved in regulating circadian rhythms [8]. Recently, a soybean root-specific WNK homolog, *GmWNK1*, which is downregulated by abscisic acid (ABA), mannitol, sucrose, polyethylene glycol, and NaCl, was shown to regulate root development, possibly by mediating ABA homeostasis in vivo [9]. Although more experiments are needed in the future, WNKs may regulate circadian rhythms through the ABA signaling pathway, as the circadian clock *LHY* gene regulates ABA accumulation and synthesis of ABA receptors, and WNKs interact with the ABA signaling pathway [10]. ABA, a plant stress hormone, regulates many abiotic stresses [11]. Therefore, several plant WNKs (WNK8 and WNK9) play important roles in ABA-dependent abiotic stress responses. AtWNK8 acts as a negative regulator of ABA signaling and regulates salt and osmotic balance by influencing the assembly of the V-ATPase complex [12,13], while *AtWNK9* acts as a positive regulator of ABA signaling and plays an important role in drought stress [14]. Moreover, plant WNKs play multiple physiological roles in regulating flowering time [15], plant longevity [16], seedling development [13], root architecture [17], and cellular pH maintenance [18]. However, the function of WNKs cells in response to biotic stress has not yet been reported. Upland cotton, a prominent fiber source in the textile industry, faces numerous biotic and abiotic stresses during production [19,20,21]. The development of cotton germplasm resources resistant to biotic and abiotic stresses has significant economic implications, and many genes, including protein kinases, play important roles in the response to these stresses [20,22]. However, the functions of WNKs in *Gossypium hirsutum* have not yet been reported.

In this study, the genome-wide annotation of WNK genes in *G. hirsutum* was performed. Specifically, we identified the members of the WNK gene family in *G. hirsutum*. The protein sequences, gene structure, upstream open reading frames (uORFs), and phylogenetic relationships of *GhWNKs* were analyzed. Moreover, the gene expression patterns of *GhWNKs* were analyzed under normal growth conditions and stress treatments, encompassing biotic stress from *Verticillium dahliae* and aphids and abiotic stresses such as cold, heat, PEG, and salt. The results revealed divergent expression patterns within the *GhWNK* gene family in upland cotton. These results suggest that *GhWNKs* function in various tissues and may play important roles in biotic and abiotic stress responses. Through further comparative analyses of the bias in homoeologous expression, our study sheds light on the biased expression pattern of *GhWNKs* pairs between the A-sub and D-sub genomes.

## 2. Results

### 2.1. The G. hirsutum Genome Contains 26 WNK Genes

According to previous studies, WNK proteins possess STKc_WNK domains [4]. Using BLASTP on the predicted proteome of *G. hirsutum* (Table S1) and confirming with NCBI Batch CD-Search, we retrieved a total of 26 coding sequences of WNK proteins. Notably, three genes (*Ghir_A12G025200.1*, *Ghir_D02G000480.1*, and *Ghir_D11G027780.1*) contained incomplete STKc_WNK domains, which were considered pseudogenes in this study (Table S2). Most of these sequences (20 out of 26) have protein lengths of 500–700 aa. The largest WNKs (*Ghir_A02G000480.1* and *Ghir_D02G003610.1*) consist of 734 aa, whereas the smallest WNK (*Ghir_A02G003230.1*) comprises only 197 aa. The molecular weights (MWs) of these amino acid sequences range from 23.07 to 83.11 kDa (Table 1). The predicted isoelectric points ranged from 4.90 to 6.89 (Table 1). Except for *Ghir_A02G003230.1*, which encodes a secreted protein, the predicted subcellular localization of most proteins encoded by *GhWNKs* was in the cytoplasm (Table 1). Moreover, the predicted instability index, aliphatic index, and grand average of hydropathicity ranged from 32.81 to 50.46, 76.44 to 90.51, and −0.63 to −0.30, respectively (Table 1), indicating that most *GhWNKs* encoded unstable hydrophilic proteins.

### 2.2. Phylogenetic Analysis of GhWNKs

**Figure 1:**
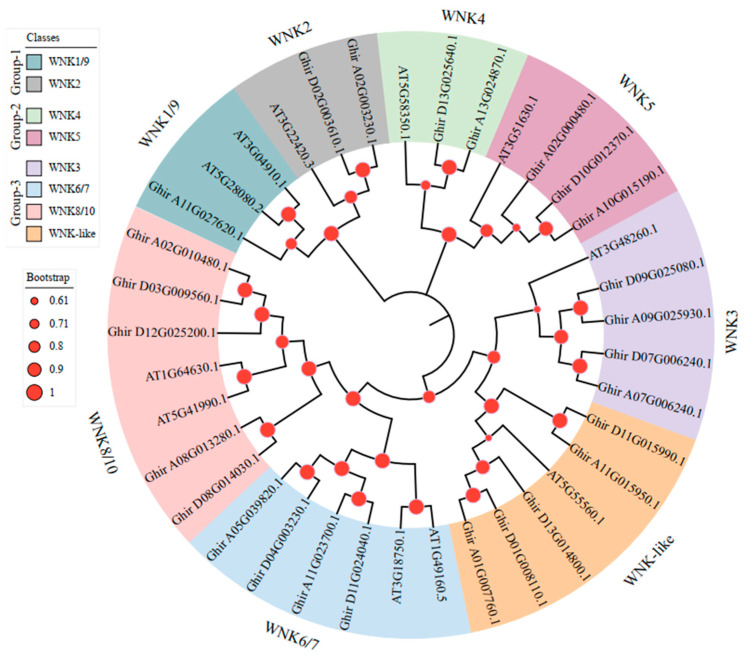
Phylogenetic tree of STKc_WNK domain-containing proteins in *G. hirsutum* and *A. thaliana*. The phylogenetic tree was constructed using MEGA-X with 1000 bootstrap replicates. The phylogenetic tree is divided into seven groups, which are shown in different colors. The bootstrap value is shown.

To better understand the evolutionary relationship between WNK genes in *G. hirsutum* and *Arabidopsis*, a phylogenetic tree was constructed using the protein sequences of 11 *AtWNKs* and 26 *GhWNKs*, and the *GhWNKs* were named based on the classification of *AtWNKs*. Phylogenetic analysis of *GhWNKs* showed that they could be divided into three groups (Figure 1). There were 3 members in group 1 (WNK1/9 with one gene each, and WNK2 with two genes), 5 members in group 2 (WNK4 with two genes, and WNK5 with three genes), and 18 members in group 3 (WNK3 with four genes, WNK6/7 with four genes each, WNK8/10 with five genes each, and WNK-like with five genes). Among them, WNK8/10 and WNK-like contained the most members, whereas WNK1/9 had the fewest, indicating that members of different WNKs have undergone different evolutionary events involving duplication and deletion. Moreover, pairwise comparisons of the 26 full-length WNK sequences revealed notable features (Figure S1). The maximum and minimum average pairwise sequence identities were determined for WNK2 and WNK5. Moreover, the degree of sequence divergence in descending order was found to be as follows: WNK5, WNK8/10, WNK-like, WNK3, WNK6/7, WNK4, and WNK2 (Figure S1). To further understand the function of WNK genes in *G. hirsutum*, the 3D structure of GhWNK proteins was predicted using SWISS-MODEL. We found that GhWNK4, GhWNK5, GhWNK6/7, GhWNK8/10, and GhWNK-like have two or more type protein structures, but GhWNK2 and GhWNK3 have only a one-type protein structure (Figure S2), suggesting that GhWNK4, GhWNK5, GhWNK6/7, GhWNK8/10, and GhWNK-like may be functionally divergent.

### 2.3. Structural and Conserved Motifs Characteristics of GhWNKs

**Figure 2:**
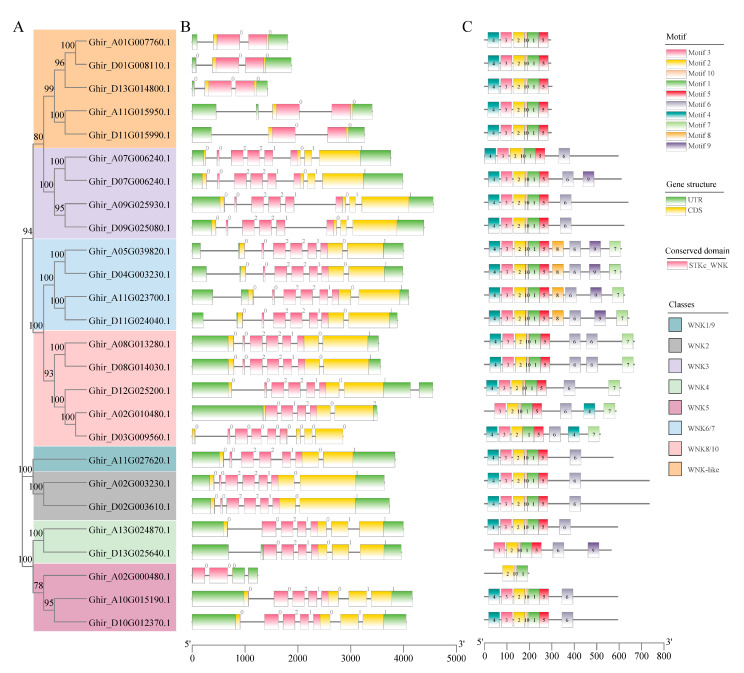
Analysis of gene structure and architecture of the conserved protein motifs in *GhWNKs*. (**A**) By using the maximum likelihood (ML) method, the phylogenetic tree was constructed based on the full-length sequences of GhWNK proteins. (**B**) Exon–intron structure of *GhWNKs*. Untranslated regions, exons, and introns are shown as light green boxes, light yellow boxes, and horizontal lines, respectively. The red boxes represent the STKc_WNK domain. (**C**) Ten types of conserved motifs, which are displayed in different colored boxes, were predicted in the GhWNK protein sequences. The sequence information for each motif is provided in Table S3.

To further understand the evolution of *GhWNK* genes, the exon–intron organization, functional domains, and conserved motifs of *GhWNKs* were uncovered. Functional domain and exon–intron organization analyses showed that all *GhWNK*s contain the STKc_WNK domain, and the number of exons is between three and nine (Figure 2A,B). MEME analysis was performed online to identify 10 conserved motifs among the 26 *GhWNK* genes (Table S3). In general, *GhWNK* motifs are similarly distributed (Figure 2C). Most members exhibit 6–8 motifs, with *Ghir_A02G000480.1* being an exception, featuring only three motifs (Figure 2C). Notably, motif 4, containing the conserved subdomain in *GhWNKs,* emphasizes the high conservation of plant WNKs, as described by Manuka et al. (2015) [4]. The only distinction observed is that the highest frequency of the amino acid at position 22 of *GhWNKs* is L, whereas that of plant WNKs is V (Figure S3).

This study uncovered variations in the uORF shape phenotypic diversity in plants [23]. However, the functions of uORFs in *GhWNKs* remain unclear. Currently, no uORF database exists for upland cotton, but one exists for *Gossypium raimondii*. Using uORFlight, uORFs were detected in 11 of the 14 WNK genes in *G.raimondii*. Additionally, 18 of the 26 *GhWNKs* were found in upland cotton (Table S4). Further analysis showed that uORFs exist in most homologous gene pairs, such as *Ghir_A11G023700*/*Ghir_D11G024040* and *Ghir_A08G013280*/*Ghir_D08G014030*. Interestingly, most uORFs were lost in one of several homologous gene pairs, such as *Ghir_D01G008110* (which lost most uORFs when paired with *Ghir_A01G007760*). These results indicate that some genes in the *GhWNK* family have undergone a loss of uORFs throughout their evolution. However, the impact on upland cotton warrants further investigation.

### 2.4. Cis-Acting Elements and Transcription Factors Were Detected in GhWNKs

**Figure 3:**
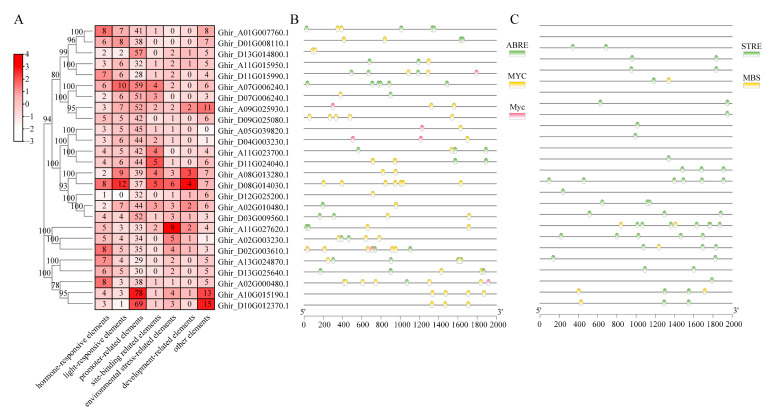
*Cis*-acting elements were predicted within the 2000 bp promoter regions of *GhWNKs*. (**A**) Schematic representation of the numbers of *cis*-acting elements detected in the promoter region of each *GhWNK*. All *cis*-acting elements were divided into seven types, and the number of *cis*-acting elements were normalized by column. (**B**,**C**) Type, quantity, and position of hormone response elements (**B**) and environmental stress-related elements (**C**) in the *GhWNK* promoter regions.

**Figure 4:**
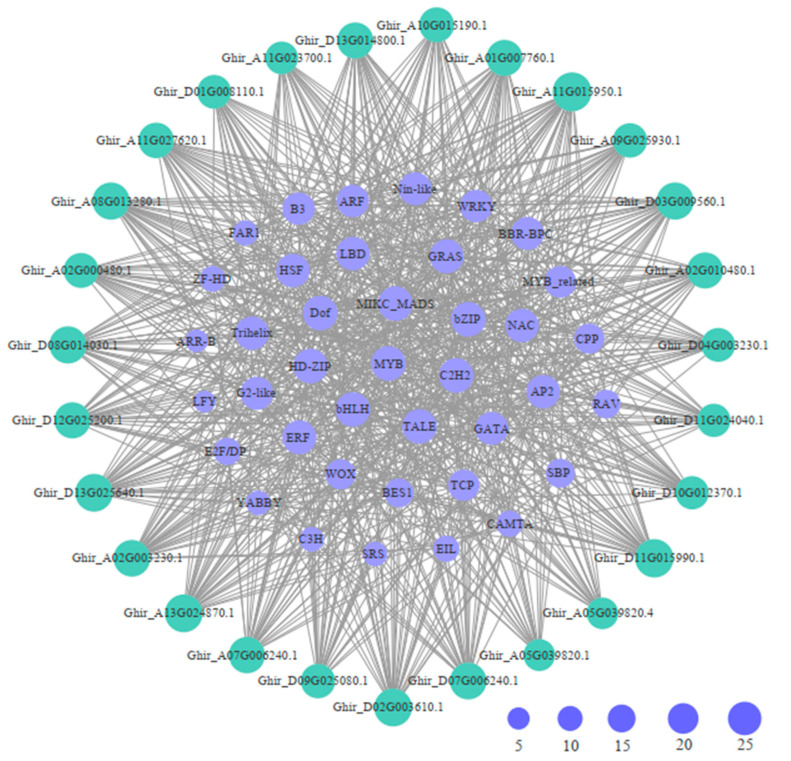
Regulatory network between *GhWNKs* and potential TFs. The green rings with gene IDs represent *GhWNKs*, the purple rings with TF names represent possible transcription factors, and the black lines represent potential regulatory relationships. The size of the rings represents the degree of potential regulatory relationships between *GhWNKs* and TFs.

To predict the *cis*-acting elements among the *GhWNKs*, the 2000 bp upstream promoter sequence of WNK genes was collected and analyzed using PlantCARE. A total of 1706 *cis*-acting elements, representing 30 types, were predicted and were divided into seven groups, including hormonal response, light response, promoter-related, site binding, environmental stress, development, and other functional categories (Table S5). There were 28–78 promoter-related elements in each *GhWNK* (Figure 3A). The 119 hormone-related components were divided into three categories, mostly related to ABA and jasmonic acid (JA) (Figure 3B). Sixty elements related to environmental stress were predicted to fall within two categories: STRE and MBS (Figure 3C). Furthermore, to comprehensively demonstrate the regulatory network of *GhWNKs* in upland cotton, possible transcription factors of *GhWNKs* were predicted. A total of 39 different types of transcription factors were predicted to be involved in regulating *GhWNK* expression (Figure 4 and Table S6). The conserved *cis*-elements, including MIKC_MADS, C2H2, TALE, bZIP, Dof, MYB, bHLH, and HD-ZIP transcription factors, were predicted to be involved in regulating *GhWNK* expression, indicating that the various *GhWNKs* are regulated by a range of 22 to 35 transcription factors. *GhWNK6/7* was predicted to be regulated by 22–26 transcription factors with minimum average values, while *GhWNK4* is regulated by 33 transcription factors with maximum average values and minimum amplitudes. *GhWNK-like* was predicted to be regulated by 26–35 transcription factors with maximum amplitudes. Moreover, the specific transcription factors LFY, CAMTA, FAR1, and YABBY were only present in *Ghir_A11G027620.1* (LFY) or *Ghir_D11G015990.1* (CAMTA, FAR1, and YABBY). These results indicate that the functions of each *GhWNK-like* species diverged during evolution.

### 2.5. Tissue-Specific Expression of GhWNKs

**Figure 5:**
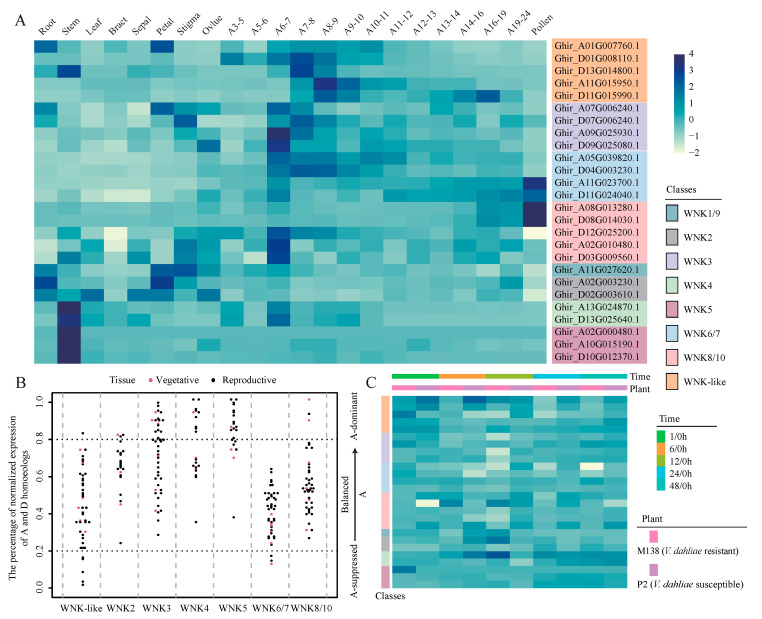
The expression pattern of *GhWNKs*. (**A**) Heatmap showing the tissue-specific expression of *GhWNKs*. (**B**) Bias in homologous gene expression within *GhWNKs* varies between tissues. (**C**) Heatmap showing the expression pattern of *GhWNKs* between *V. dahlia*-resistant and -susceptible cotton cultivar after infection with V991. A3–5 represents anthers in 3–5 mm bud, A5–6 represents anthers in 5–6 mm bud, and so on.

To comprehensively understand the functions of *GhWNKs*, spatial and temporal expression analyses were conducted on all *GhWNKs* using RNA-seq data from various tissues, including root, stem, leaf, bract, petal, stigma, ovule, different stages of anther, and pollen (Figure 5A and Table S7). The results showed that *GhWNK3* and *GhWNK-like* are preferentially expressed in the anther tetrad or mononuclear stage, whereas *GhWNK6/7* and *GhWNK8/10* (*Ghir_A08G013280.1* and *Ghir_D08G014030.1*) are preferentially expressed in other developmental stages or pollen. Further analysis demonstrated a ubiquitous gene expression pattern in *GhWNK8/10* (*Ghir_D12G025200.1*, *Ghir_A02G010480.1*, and *Ghir_D03G009560.1*), *GhWNK1*, *GhWNK2*, and *GhWNK4*. Moreover, *GhWNK5* was found to be specifically expressed in the stem.

Interestingly, there was variability in homoeologous expression bias across tissues in tetraploid upland cotton within the *GhWNKs*. To determine patterns of homoeologous expression within *GhWNKs*, the distribution of *GhWNKs* on the *G. hirsutum* chromosomes was examined. It was revealed that *GhWNKs* were unevenly localized on 26 *G. hirsutum* chromosomes (Figure S4). The gene pairs were then identified using collinearity analysis, indicating 15 gene pairs within *GhWNKs* and three pseudogenes of WNKs, among which *Ghir_A01G007760.1* formed 2 gene pairs with *Ghir_D01G008110.1* and *Ghir_D13G014800.1* (Figure S4). Finally, 11 gene pairs were selected for studying the homologous expression bias of *GhWNKs*, excluding gene pairs with pseudogenes and *Ghir_D13G014800.1*. The categories of A-suppressed and A-dominant were assigned based on the abundance of transcripts in subgenome D compared with subgenome A. The results showed that *GhWNK2* and *GhWNK8/10* were mainly balanced in vegetative and reproductive tissues but were dominant in several vegetative and reproductive tissues. *GhWNK-like* was mainly balanced in the vegetative and reproductive tissues, whereas A-dominant or A-suppressed cells were found in several vegetative and reproductive tissues. The percentages of A-dominant and balanced expression in *GhWNK3*, *GhWNK4*, and *GhWNK5* were similar in both vegetative and reproductive tissues. *GhWNK6/7* was mainly balanced in vegetative and reproductive tissues but was suppressed in several vegetative and reproductive tissues (Figure 5B).

Furthermore, to investigate the evolutionary process, the Ka/Ks ratios of *GhWNK* homoeologous pairs were computed as indicators of selection pressure. The results showed that the Ka/Ks ratios of *GhWNK* homoeologous pairs ranged from 0.1474 to 0.7273 (Table S8), suggesting that the *GhWNK* homoeologous pairs were strongly purified during evolution, particularly the *GhWNK4* and *GhWNK-like* homoeologous pairs.

### 2.6. Expression of GhWNKs under Biotic and Abiotic Stress

To investigate whether *GhWNKs* participate in the response to *V. dahliae* and *Aphis*, the expression patterns of *GhWNK* infiltration with *V.dahliae* (V991) and *Aphis gossypii* were collected. The results showed that *GhWNK2* (*Ghir_A02G003230.1*), *GhWNK3* (*Ghir_A07G006240.1* and *Ghir_A09G025930.1*), *GhWNK4* (*Ghir_A13G024870.1* and *Ghir_D13G025640.1*), *GhWNK5* (*Ghir_A02G000480.1*), *GhWNK6/7* (*Ghir_A05G039820.1* and *Ghir_D04G003230.1*), *GhWNK8/10* (*Ghir_D08G014030.1* and *Ghir_A02G010480.1*), and *GhWNK-like* (*Ghir_A01G007760.1*, *Ghir_D01G008110.1*, and *Ghir_D13G014800.1*) exhibited differential expression among *V. dahliae*-resistant and -susceptible cotton cultivars after infection with V991 at one or more time points (including 1 h, 3 h, 6 h, 12 h, 24 h, and 48 h). Among them, the most differentially expressed genes were found at 6 h in the *V. dahlia*-resistant cotton cultivar after infection with V991. In particular, the expression of *Ghir_A05G039820.1* (*GhWNK6/7*) was downregulated at 6 h, 12 h, and 24 h in the *V. dahlia*-resistant cotton cultivar after infection with V991, while it was not differentially expressed in the susceptible cotton cultivar (Figure 5C and Table S9). This indicates that *Ghir_A05G039820.1* may act as a negative regulator in response to *V. dahliae*. Expression pattern analysis of *GhWNKs* after infection with *A. gossypii* showed that only one gene (*Ghir_D11G015990.1*) was differentially expressed between *A. gossypii*-resistant and -susceptible cotton cultivars (Table S10), suggesting that *Ghir_D11G015990.1* may participate in the stress response to *A. gossypii*. These results suggest that *GhWNKs* (particularly *Ghir_A05G039820.1*) are differentially expressed in response to *V. dahliae* and may be important in the upland cotton response to *V. dahliae*, but not in response *to A. gossypii*. However, more experiments are needed in the future to confirm these findings.

Furthermore, to investigate the function of *GhWNKs* under abiotic stress, the expression patterns under cold (4 ℃), heat (37 ℃), PEG (200 g/liter), and salt (0.4 M) stress were obtained, showing that the expression of *GhWNK* gene family members changed significantly under abiotic stress (Figure S5). *GhWNK1/9*, *GhWNK2*, *GhWNK4*, *GhWNK8/10* (*Ghir_A08G013280.1* and *Ghir_D08G014030.1*), and *GhWNK-like* (*Ghir_A01G007760.1* and *Ghir_D01G008110.1*) were upregulated under cold stress. Interestingly, these genes are distributed in different evolutionary branches, which suggests that the function of the *GhWNK* gene family in response to cold is highly conserved. Therefore, these genes can serve as important candidates for molecular mechanisms in response to cold. Expression under heat stress was characterized by the presence of a large number of genes that were upregulated at 1 h and downregulated at 24 h. These genes overlap with those that respond to cold conditions. A large number of genes were downregulated under PEG stress, while *Ghir_D02G003610.1* (*GhWNK2*) was upregulated under PEG stress at 12 h and 24 h. Expression patterns under PEG and salt stress were similar. However, there were exceptions; for example, *GhWNK4* was downregulated under PEG, but upregulated under salt stress at 3 h. Based on these results, *GhWNKs* (particularly *GhWNK4*) may also play a crucial role during abiotic stress, which is consistent with previous studies [2].

## 3. Discussion

### 3.1. The Conserved Motif and Sequence Characterization of GhWNKs

This family of WNKs has been identified and characterized in several plants, including *Arabidopsis* [3], *Oryza* [4], *Glycine max* [24], *Bambusoideae* [25], and *Prunus persica* [26]. In this study, we identified 26 WNKs encoding unstable hydrophilic proteins in upland cotton and revealed that their structural domains were conserved through phylogenetic tree, gene structure, and conserved motif analyses (Figure 2). The conserved motif of *GhWNKs* is Gly X-Gly X-X-Lys-X-Val in kinase subdomain-1, with an unusual position of the lysine (K) residue. This motif replaces Gly-X-GIy-X-X-Gly-X-Val in the kinase domain and forms the single active center of *GhWNKs*, consistent with previous studies [4]. Three-dimensional structural analysis of these conserved motifs will facilitate investigation of the catalytic mechanism of WNK and enable the identification of potential catalytic substrates in the future.

Members of the WNK family have been shown to play key roles in the circadian cycle and abiotic stress responses [27]. Therefore, to investigate the sequence characterization of *GhWNKs*, the *cis*-acting elements in the promoters were identified. The promoter of *Ghir_D08G014030.1* (*GhWNK8/10*) exhibited the lightest response *cis*-acting element, while hormonal response *cis*-acting elements, including ABA responsive element (ABRE), dehydration responsive elements (MBS), and MYC, were also identified in *GhWNKs* (Figure 3 and Table S5). In particular, the G-box, a conserved circadian motif, was identified in 14 out of 26 *GhWNKs* (Table S5). Furthermore, the MIKC_MADS, C2H2, TALE, bZIP, Dof, MYB, bHLH, and HD-ZIP transcription factors were the most abundant in *GhWNKs* (Figure 4 and Table S6). Based on these data, this study highlights *cis*-acting elements such as light response, hormonal response, circadian rhythm, and the regulatory network of *GhWNKs* in upland cotton. These results shed light on the functions of *GhWNKs*.

### 3.2. Functions of GhWNK Genes in Abiotic and Biotic Stresses

Crop growth is accompanied by a variety of biotic and abiotic stresses [28,29]. Cotton, an important cash crop, is exposed to a number of abiotic and biotic stresses, such as drought, heat, Verticillium wilt, and aphids [30,31,32,33]. WNK genes, such as salt and osmotic stress genes, play important roles in abiotic stress [2]. Molecular mechanism studies have demonstrated a crosstalk between plant WNKs and ABA under abiotic stress [14]. The expression patterns of several *GhWNKs* (particularly *GhWNK4*) are induced by abiotic stresses including cold (4 °C), heat (37 °C), PEG (200 g/L), and salt (0.4 M), suggesting that *GhWNKs* may also play important roles in abiotic stress, which is consistent with previous studies [2]. Interestingly, the promoter sequence of *GhWNK4* is enriched in hormone response elements. This may explain the function of *GhWNK4* in abiotic stress, as hormones, such as ABA, play important roles in abiotic stress. However, studies on the role of WNK in response to biotic stress are limited. In this study, based on the RNA data of cotton infiltration with *V. dahliae* (V991) and *A. gossypii*, the role of *GhWNKs* may be important in the response of upland cotton to *V. dahliae*, but not to *A. gossypii*. This provides a research direction for functional studies of the resistance mechanism to Verticillium wilt. Verticillium wilt is a destructive disease that causes significant cotton losses [34]. Many studies have shown that cysteine-rich receptor-like kinases (CRKs), JA, and salicylic acid signaling pathways are involved in regulating defense responses to *V. dahliae* [35,36,37]. However, research on cotton resistance genes in response to *V. dahliae* is still limited, and cotton germplasm resources resistant to *V. dahliae* remain scarce [34]. Investigating the functions of *GhWNKs* as protein kinases in response to *V. dahliae* will be helpful in breeding cotton that is resistant to Verticillium wilt. Furthermore, in future works, it will be important to study the catalytic substrates of *GhWNKs* to understand their underlying molecular mechanisms.

### 3.3. The Bias Expression Patterns of GhWNKs Homologous Gene Pairs

Polyploidy is a common plant phenomenon. It has been proposed that it confers adaptive plasticity and shape evolution [38], facilitating the domestication and adaptation of several major crop species [39]. In polyploids, gene duplication alters the transcriptional landscape, and a bias in the expression of homoeologous genes has been observed. For example, the bias in homoeologous expression varies between tissues, with approximately 30% of wheat homeologs showing unbalanced expression in wheat [38]. Bias in the expression of homologous genes is also observed in cotton [40]. However, the role of bias in the homologous expression of *GhWNKs* remains unclear. In this study, the bias in homoeologous expression within *GhWNKs* varied among tissues observed in upland cotton. The percentage of A-dominant cells surpassed that of A-suppressed cells (Figure 5B). This suggests that the A-dominant expression pattern may play an important role in its function. Therefore, WNK genes in the A-sub genome have been highlighted in gene function studies, offering a framework to assist researchers and breeders in developing strategies to improve cotton traits by manipulating individual or multiple homeologs. However, the function of bias in homoeologous expression varies in *GhWNKs*, and the mechanism of its generation requires further investigation.

## 4. Materials and Methods

### 4.1. Identification of the WNK Kinase Gene Family in Upland Cotton

To identify the WNK kinase gene family in *G. hirsutum*, the predicted WNK protein sequence in upland cotton was obtained using InterPro (http://www.ebi.ac.uk/interpro/, accessed on 10 May 2023). The candidate WNK protein sequence was screened using BLAST in Cottongen (https://www.cottongen.org/, accessed on 10 May 2023), using the upland cotton TM-1 genome [41]. The NCBI Batch CD-Search function (https://www.ncbi.nlm.nih.gov/Structure/bwrpsb/bwrpsb.cgi/, accessed on 10 May 2023) was used to confirm whether the candidate WNK genes had the characteristic STKc_WNK domains (NCBI, cd13983). Genes with incomplete STKc_WNK domains were eliminated. The protein length (pI) and MW of all candidate WNK genes in *G. hirsutum* were determined using ExPASy (https://www.arabidopsis.org/, accessed on 10 May 2023) [42] via TBtools V2.012 [43], and their subcellular localization was predicted using Plant-mPLoc(http://www.csbio.sjtu.edu.cn/bioinf/plant-multi/, accessed on 10 May 2023) [44].

### 4.2. Phylogenetic Analysis and Molecular Evolution Analyses

The WNK gene family sequences of *G. hirsutum* and *A. thaliana* were obtained from Cottongen and TAIR (https://www.arabidopsis.org/, accessed on 10 May 2023), respectively. A multiple sequence alignment of the full-length protein sequences of the *AtWNKs* and *GhWNKs* gene family was performed using Clustal-X 1.8 [45]. A phylogenetic tree was constructed using the maximum likelihood (ML) method, incorporating options for gamma distribution, the Jones, Taylor, and Thornton amino acid substitution model, and 1000 bootstrap replicates in MEGA-X (https://www.megasoftware.net/, accessed on 10 May 2023). Finally, the tree was annotated and identified using iTOL software (https://itol.embl.de/, accessed on 10 May 2023) [46].

Synonymous substitution (*Ks*) rates and non-synonymous substitutions (*Ka*) among *GhWNKs* pairwise were calculated using the PAML package v4.10.6 [6,47].

### 4.3. Analysis of Conserved Motifs, Gene Structure, Functional Domains, 3D Structure, and uORFs

The conserved motifs of the *GhWNKs* gene family were predicted using the MEME program [48], with default algorithm parameters. The maximum number of motifs was set to 10. The GFF3 data of *GhWNKs* proteins and the reference genome of *G. hirsutum* [36] were downloaded from Cottongen. The gene structure and functional domains were analyzed and visualized using NCBI Batch CD-Search [49,50] and TBtools, respectively. To obtain the 3D structure of the GhWNK protein, the GhWNK protein sequence was submitted to SWISS-MODEL (https://swissmodel.expasy.org, accessed on 10 May 2023/) using the default algorithm parameters. The uORFs of WNKs were detected using uORFlight (http://www.rnairport.com:443/Tool_uORFFinder.php/, accessed on 10 May 2023) [51]. The uORFs of *GhWNKs* were detected using sequences for ICCu (initiation codon context for upstream open reading frames) and ICCm (initiation codon context for major open reading frames).

### 4.4. Promoter cis-Acting Elements and TFs Prediction

To identify the promoter *cis*-acting elements of *GhWNKs*, the promoter sequences of *GhWNKs* with 2000 bp were obtained, predicted using PlantCARE (http://bioinformatics.psb.ugent.be/webtools/plantcare/html/, accessed on 10 May 2023) [52], and visualized using TBtools. The transcription factors of *GhWNKs* were predicted using PlantRegMap(http://plantregmap.gao-lab.org/go.php/, accessed on 10 May 2023) [53], with *A. thaliana* as the target species. Cytoscape 3.6.0 was used to visualize the relationship between transcription factors and *GhWNKs* [54].

### 4.5. Gene Expression Analysis Based on RNA-Seq and Digital Gene Expression Data

Tissue expression data for *GhWNKs* were provided by Zhang et al. (2022) [55] and visualized using R (https://www.r-project.org/, accessed on 10 May 2023). These data were normalized to different genes and hierarchically clustered based on the phylogenetic tree. Homologous gene expression bias of *GhWNKs* was determined, as described by Ramírez-González et al. (2018) [38]. To standardize the relative expression of each homeolog across gene pairs, we normalized the absolute FPKM for each gene within the gene pairs as follows:

expression*_A_* = FPKM(*A*)/[FPKM(*A*) + FPKM(*D*)]; expression*_D_* = FPKM(*D*)/[FPKM(*A*) + FPKM(*D*)] (1)

where *A* and *D* represent the genes corresponding to the A and D subgenome homeologs in gene pairs. The percentages of homologous gene expression in the A-sub and D-sub genomes were calculated and visualized using R. Moreover, expression patterns following infiltration with *V. dahliae* (V991) and *A. gossypii* were obtained [31,56]. Notably, M138 represented a *V. dahlia*-resistant *G. hirsutum* cultivar, while P2 was a *V. dahlia*-susceptible cotton cultivar derived from the MAGIC population. Xinluzao 61 is an aphid-resistant *G. hirsutum* cultivar, while Xinluzao 50 is an aphid-susceptible *G. hirsutum* cultivar. The expression patterns under cold (4 °C), heat (37 °C), PEG (200 g/liter), and salt (0.4 M) stress were derived from CottonFGD (https://cottonfgd.net/, accessed on 10 May 2023) [57]. Expression patterns were visualized using R.

## Figures and Tables

**Table 1:** Detailed information of the 26 predicted GhWNK proteins in *G. hirsutum*.

Gene ID	Gene Name	Gene Location	Strand	CDS	Protein Length (aa)	Amino Acids MW (kDa)	PI	Loctree3	Instability Index	Aliphatic Index	Grand Average of Hydropathicity
*Ghir_A01G007760.1*	*GhWNK-like*	Ghir_A01:12971108–12972916	−	882	294	33.51	5.28	cytoplasm	32.81	83.5	−0.471
*Ghir_D01G008110.1*	*GhWNK-like*	Ghir_D01:11157645–11159519	−	885	295	33.70	5.28	cytoplasm	34.52	83.9	−0.47
*Ghir_D13G014800.1*	*GhWNK-like*	Ghir_D13:48337800–48339222	+	1692	564	63.88	6.37	cytoplasm	47.56	81.06	−0.483
*Ghir_A11G015950.1*	*GhWNK-like*	Ghir_A11:18534787–18538191	+	1719	573	65.83	4.96	cytoplasm	42.57	76.88	−0.557
*Ghir_D11G015990.1*	*GhWNK-like*	Ghir_D11:15838773–15842033	+	1929	643	72.09	5.55	cytoplasm	46.74	82.94	−0.402
*Ghir_A07G006240.1*	*GhWNK3*	Ghir_A07:7499395–7503152	+	1785	595	66.98	5.49	cytoplasm	41.77	83.87	−0.525
*Ghir_D07G006240.1*	*GhWNK3*	Ghir_D07:6786435–6790420	+	1827	609	68.64	5.17	cytoplasm	41.34	78.1	−0.628
*Ghir_A09G025930.1*	*GhWNK3*	Ghir_A09:81714196–81718754	−	1917	639	72.13	5.29	cytoplasm	43.48	86.67	−0.528
*Ghir_D09G025080.1*	*GhWNK3*	Ghir_D09:52477764–52482145	−	1863	621	69.96	5.22	cytoplasm	44.82	85.89	−0.548
*Ghir_A05G039820.1*	*GhWNK6/7*	Ghir_A05:104554042–104558039	−	1830	610	68.01	5.25	cytoplasm	45.82	88.07	−0.362
*Ghir_D04G003230.1*	*GhWNK6/7*	Ghir_D04:4406163–4410147	+	1827	609	67.81	5.25	cytoplasm	44.7	87.9	−0.376
*Ghir_A11G023700.1*	*GhWNK6/7*	Ghir_A11:71644048–71648140	+	891	297	33.68	5.89	cytoplasm	39.07	87.27	−0.328
*Ghir_D11G024040.1*	*GhWNK6/7*	Ghir_D11:43781586–43785463	+	891	297	33.70	5.81	cytoplasm	37.84	88.59	−0.304
*Ghir_A08G013280.1*	*GhWNK8/10*	Ghir_A08:95894478–95898002	−	2001	667	75.44	4.92	cytoplasm	47.98	83.01	−0.41
*Ghir_D08G014030.1*	*GhWNK8/10*	Ghir_D08:48419558–48423120	−	2004	668	75.62	4.96	cytoplasm	45.85	83.19	−0.418
*Ghir_D12G025200.1*	*GhWNK8/10*	Ghir_D12:59173626–59178177	+	1821	607	68.55	5.01	cytoplasm	41.94	84.83	−0.404
*Ghir_A02G010480.1*	*GhWNK8/10*	Ghir_A02:44867018–44870515	+	1758	586	66.39	4.94	cytoplasm	45.49	84.16	−0.371
*Ghir_D03G009560.1*	*GhWNK8/10*	Ghir_D03:33375034–33377884	+	1548	516	58.96	4.9	cytoplasm	45.57	82.95	−0.456
*Ghir_A11G027620.1*	*GhWNK1/9*	Ghir_A11:108616362–108620199	+	1872	624	70.39	6	cytoplasm	50.46	83.61	−0.402
*Ghir_A02G003230.1*	*GhWNK2*	Ghir_A02:3714173–3717808	+	591	197	23.07	6.89	secreted	41.99	90.51	−0.295
*Ghir_D02G003610.1*	*GhWNK2*	Ghir_D02:4679685–4683413	+	2202	734	82.80	5.07	cytoplasm	45.16	81.4	−0.497
*Ghir_A13G024870.1*	*GhWNK4*	Ghir_A13:108318875–108322870	+	1779	593	67.41	6.2	cytoplasm	45.42	78.25	−0.535
*Ghir_D13G025640.1*	*GhWNK4*	Ghir_D13:63297294–63301250	+	900	300	34.30	5.29	cytoplasm	40.34	80.87	−0.513
*Ghir_A02G000480.1*	*GhWNK5*	Ghir_A02:293503–294742	+	2202	734	83.11	5.03	cytoplasm	45.31	81.01	−0.515
*Ghir_A10G015190.1*	*GhWNK5*	Ghir_A10:83087865–83092029	−	1779	593	67.69	5.31	cytoplasm	41.81	76.44	−0.487
*Ghir_D10G012370.1*	*GhWNK5*	Ghir_D10:19346669–19350715	+	1779	593	67.72	5.36	cytoplasm	42.28	76.93	−0.47

## Data Availability

All data generated or analyzed during this study are included in this published article and its additional files. The datasets used and analyzed in the current study are available from the corresponding author upon reasonable request.

## Supplementary Materials

The following supporting information can be downloaded at: https://www.mdpi.com/article/10.3390/plants12234036/s1, Figure S1: Pairwise sequence identity of full-length GhWNK proteins; Figure S2: 3D structures of the GhWNK proteins. The 3D structures of GhWNK-like (A), GhWNK3 (B), GhWNK6/7 (C), GhWNK8/10 (D), GhWNK1/9 (E), GhWNK2 (F), GhWNK4 (G), and GhWNK5 (H); Figure S3: Conserved GhWNK amino acid subdomain in motif 4. Graphical representation of the conserved GhWNK amino acids subdomain-1 and -2 obtained using the MEME tool (https://meme-suite.org/meme/tools/meme, accessed on 10 May 2023). The overall height of the stack indicates the sequence conservation at that position, whereas the height of the symbols within the stack indicates the relative frequency of each amino acid at that position. The lower and upper horizontal arrows show the N-terminal conserved signature of the glycine-rich stretch and protein kinase subdomain-1 and -2, respectively. The catalytic lysine (K) residue of subdomain-2 was replaced by asparagine (N)/serine (S) at position 30 and shifted to subdomain-1 at position 13; Figure S4: Genomic localization and collinearity analysis of *GhWNKs* and three pseudogenes. Green triangles represent the pseudogenes of *GhWNKs*; Figure S5: Expression patterns of *GhWNKs* under cold, heat, PEG, and salt stress, Table S1: The conservative domain prediction results of the partial WNK fragments in Gossypium hirsutum, Table S2: List of ZmDIRs CDS and protein sequences, Table S3: The MEME Motif Sequence and Length of WNK in Gossypium hirsutum, Table S4: Prediction of uORF from WNK genes in Gossypium raimondii and Gossypium hirsutum, Table S5: Cis-acting elements of 2000bp upstream sequence of WNK genes in Gossypium hirsutum, Table S6: Potential transcription factors of WNK in Gossypium hirsutum, Table: The tissue expression pattern of GhWNK, Table S8: The Ka/Ks ratios of *GhWNKs* homoeolog pairs, Table S9: The FPKM value of *GhWNKs* in M138 and P2 infiltration with V.dahliae (V991), Table S10: The FPKM value of *GhWNKs* in Xinluzao-50 and Xinluzao-61 infiltration with Aphis gossypii.

## References

[1] Shekarabi M., Zhang J.W., Khanna A.R., Ellison D.H., Delpire E., Kahle K.T. (2017). WNK Kinase Signaling in Ion Homeostasis and Human Disease. Cell Metab..

[2] Saddhe A.A., Karle S.B., Aftab T., Kumar K. (2021). With no lysine kinases. the key regulatory networks and phytohormone cross talk in plant growth, development and stress response. Plant Cell Rep..

[3] Wang Y., Liu K., Liao H., Zhuang C., Ma H., Yan X. (2008). The plant WNK gene family and regulation of flowering time in Arabidopsis. Plant Biol..

[4] Manuka R., Saddhe A.A., Kumar K. (2015). Genome-wide identification and expression analysis of WNK kinase gene family in rice. Comput. Biol. Chem..

[5] Li L., Zhang C., Huang J., Liu Q., Wei H., Wang H., Liu G., Gu L., Yu S. (2021). Genomic analyses reveal the genetic basis of early maturity and identification of loci and candidate genes in upland cotton (*Gossypium hirsutum* L.). Plant Biotechnol. J..

[6] Li X., Wu Y., Chi H., Wei H., Wang H., Yu S. (2022). Genomewide Identification and Characterization of the Genes Involved in the Flowering of Cotton. Int. J. Mol. Sci..

[7] Kumar K., Rao K.P., Biswas D.K., Sinha A.K. (2011). Rice WNK1 is regulated by abiotic stress and involved in internal circadian rhythm. Plant Signal. Behav..

[8] Matsushika A., Imamura A., Yamashino T., Mizuno T. (2002). Aberrant Expression of the Light-Inducible and Circadian-Regulated APRR9 Gene Belonging to the Circadian-Associated APRR1/TOC1 Quintet Results in the Phenotype of Early Flowering in Arabidopsis thaliana. Plant Cell Physiol..

[9] Wang Y., Suo H., Zheng Y., Liu K., Zhuang C., Kahle K.T., Ma H., Yan X. (2010). The soybean root-specific protein kinase GmWNK1 regulates stress-responsive ABA signaling on the root system architecture. Plant J..

[10] Singh M., Mas P. (2018). A Functional Connection between the Circadian Clock and Hormonal Timing in Arabidopsis. Genes.

[11] Berens M.L., Wolinska K.W., Spaepen S., Ziegler J., Nobori T., Nair A., Krüler V., Winkelmüller T.M., Wang Y., Mine A. (2019). Balancing trade-offs between biotic and abiotic stress responses through leaf age-dependent variation in stress hormone cross-talk. Proc. Natl. Acad. Sci. USA.

[12] Zhang B.G., Liu K.D., Zheng Y., Wang Y.X., Wang J.X., Liao H. (2013). Disruption of AtWNK8 Enhances Tolerance of Arabidopsis to Salt and Osmotic Stresses via Modulating Proline Content and Activities of Catalase and Peroxidase. Int. J. Mol. Sci..

[13] Waadt R., Jawurek E., Hashimoto K., Li Y., Scholz M., Krebs M., Czap G., Hong-Hermesdorf A., Hippler M., Grill E. (2019). Modulation of ABA responses by the protein kinase WNK8. FEBS Lett..

[14] Xie M., Wu D., Duan G., Wang L., He R., Li X., Tang D., Zhao X., Liu X. (2014). AtWNK9 is regulated by ABA and dehydration and is involved in drought tolerance in Arabidopsis. Plant Physiol. Biochem..

[15] Urano D., Czarnecki O., Wang X., Jones A.M., Chen J.-G. (2015). Arabidopsis Receptor of Activated C Kinase1 Phosphorylation by with no lysine8 kinase1 open. Plant Physiol..

[16] Lo S., Fatokun C., Boukar O., Gepts P., Close T.J., Munoz-Amatriain M. (2020). Identification of QTL for perenniality floral scent in cowpea (*Vigna unguiculata*, L. Walp.). PLoS ONE.

[17] Wang P.C., Du Y.Y., Li Y.A., Ren D.T., Song C.P. (2010). Hydrogen Peroxide-Mediated Activation of MAP Kinase 6 Modulates Nitric Oxide Biosynthesis and Signal Transduction in Arabidopsis. Plant Cell.

[18] Hong-Hermesdorf A., Brux A., Gruber A., Gruber G., Schumacher K. (2006). A WNK kinase binds and phosphorylates V-ATPase subunit C. FEBS Lett..

[19] Hu Y., Chen J., Fang L., Zhang Z., Ma W., Niu Y., Ju L., Deng J., Zhao T., Lian J. (2019). Gossypium barbadense and Gossypium hirsutum genomes provide insights into the origin and evolution of allotetraploid cotton. Nat. Genet..

[20] Chen L., Sun H., Wang F., Yue D., Shen X., Sun W., Zhang X., Yang X. (2020). Genome-wide identification of MAPK cascade genes reveals the GhMAP3K14–GhMKK11–GhMPK31 pathway is involved in the drought response in cotton. Plant Mol. Biol..

[21] Qiu P., Li J., Zhang L., Chen K., Shao J., Zheng B., Yuan H., Qi J., Yue L., Hu Q. (2023). Polyethyleneimine-coated MXene quantum dots improve cotton tolerance to Verticillium dahliae by maintaining ROS homeostasis. Nat. Commun..

[22] An Q., Pan Z., Aini N., Han P., Wu Y., You C., Nie X. (2023). Identification of candidate genes for aphid resistance in upland cotton by QTL mapping and expression analysis. Crop J..

[23] Wang J., Liu J., Guo Z. (2023). Natural uORF variation in plants. Trends Plant Sci..

[24] Wang Y., Suo H., Zhuang C., Ma H., Yan X. (2011). Overexpression of the soybean GmWNK1 altered the sensitivity to salt and osmotic stress in Arabidopsis. J. Plant Physiol..

[25] Liu R., Vasupalli N., Hou D., Stalin A., Wei H., Zhang H., Lin X. (2022). Genome-wide identification and evolution of WNK kinases in Bambusoideae and transcriptional profiling during abiotic stress in Phyllostachys edulis. PeerJ.

[26] Cao S.H., Hao P.P., Shu W.S., Wang G.M., Xie Z.H., Gu C., Zhang S.L. (2019). Phylogenetic and Expression Analyses of With-No-Lysine Kinase Genes Reveal Novel Gene Family Diversity in Fruit Trees. Hortic. Plant J..

[27] Nakamichi N., Murakami-Kojima M., Sato E., Kishi Y., Yamashino T., Mizuno T. (2002). Compilation and characterization of a novel WNK family of protein kinases in Arabiodpsis thaliana with reference to circadian rhythms. Biosci. Biotechnol. Biochem..

[28] Zhu J.K. (2016). Abiotic Stress Signaling and Responses in Plants. Cell.

[29] Ngou B.P.M., Ding P., Jones J.D.G. (2022). Thirty years of resistance. Zig-zag through the plant immune system. Plant Cell.

[30] Zhang Y., Chen B., Sun Z., Liu Z., Cui Y., Ke H., Wang Z., Wu L., Zhang G., Wang G. (2021). A large-scale genomic association analysis identifies a fragment in Dt11 chromosome conferring cotton Verticillium wilt resistance. Plant Biotechnol. J..

[31] Li W., Mi X., Jin X., Zhang D., Zhu G., Shang X., Zhang D., Guo W. (2022). Thiamine functions as a key activator for modulating plant health and broad-spectrum tolerance in cotton. Plant J..

[32] Khan A.H., Wu Y., Luo L., Ma Y., Li Y., Ma H., Luo A., Zhang R., Zhu L., Lin Y. (2022). Proteomic analysis reveals that the heat shock proteins 70-17 and BiP5 enhance cotton male fertility under high-temperature stress by reducing the accumulation of ROS in anthers. Ind. Crops Prod..

[33] Hu W., Liu Y., Loka D.A., Zahoor R., Wang S., Zhou Z. (2019). Drought limits pollen tube growth rate by altering carbohydrate metabolism in cotton (*Gossypium hirsutum*) pistils. Plant Sci. Int. J. Exp. Plant Biol..

[34] Sun L., Zhu L., Xu L., Yuan D., Min L., Zhang X. (2014). Cotton cytochrome P450 CYP82D regulates systemic cell death by modulating the octadecanoid pathway. Nat. Commun..

[35] Zhao J., Sun Y., Li X., Li Y. (2022). Cysteine-Rich Receptor-Like Kinase5 (CRK5) and CRK22 regulate the response to Verticillium dahliae toxins. Plant Physiol..

[36] Hu Q., Min L., Yang X., Jin S., Zhang L., Li Y., Ma Y., Qi X., Li D., Liu H. (2018). Laccase GhLac1 Modulates Broad-Spectrum Biotic Stress Tolerance via Manipulating Phenylpropanoid Pathway and Jasmonic Acid Synthesis. Plant Physiol..

[37] Gao W., Long L., Zhu L.-F., Xu L., Gao W.-H., Sun L.-Q., Liu L.-L., Zhang X.-L. (2013). Proteomic and Virus-induced Gene Silencing (VIGS) analyses reveal that gossypol, brassinosteroids, and jasmonic acid contribute to the resistance of cotton to Verticillium dahliae. Mol. Cell. Proteom..

[38] Ramirez-Gonzalez R.H., Borrill P., Lang D., Harrington S.A., Brinton J., Venturini L., Davey M., Jacobs J., van Ex F., Pasha A. (2018). The transcriptional landscape of polyploid wheat. Science.

[39] Akagi T., Jung K., Masuda K., Shimizu K.K. (2022). Polyploidy before and after domestication of crop species. Curr. Opin. Plant Biol..

[40] Wang M., Tu L., Lin M., Lin Z., Wang P., Yang Q., Ye Z., Shen C., Li J., Zhang L. (2017). Asymmetric subgenome selection and cis-regulatory divergence during cotton domestication. Nat. Genet..

[41] Wang M., Tu L., Yuan D., Zhu D., Shen C., Li J., Liu F., Pei L., Wang P., Zhao G. (2019). Reference genome sequences of two cultivated allotetraploid cottons, Gossypium hirsutum and Gossypium barbadense. Nat. Genet..

[42] Wilkins M.R., Gasteiger E., Bairoch A., Sanchez J.C., Hochstrasser D.F. (1999). Protein Identification and Analysis Tools in the ExPASy Server. Methods Mol. Biol..

[43] Chen C., Chen H., Zhang Y., Thomas H.R., Frank M.H., He Y., Xia R. (2020). TBtools. An Integrative Toolkit Developed for Interactive Analyses of Big Biological Data. Mol. Plant.

[44] Chou K.-C., Shen H.-B. (2010). Plant-mPLoc. a top-down strategy to augment the power for predicting plant protein subcellular localization. PLoS ONE.

[45] Larkin M. (2007). Clustal W and Clustal X v. 2.0. Bioinformatics.

[46] Letunic I., Bork P. (2006). Interactive Tree Of Life (iTOL). an online tool for phylogenetic tree display and annotation. Bioinformatics.

[47] Yang Z. (2007). PAML 4. phylogenetic analysis by maximum likelihood. Mol. Biol. Evol..

[48] Bailey T.L., Johnson J., Grant C.E., Noble W.S. (2015). The MEME Suite. Nucleic Acids Res..

[49] Marchler-Bauer A., Derbyshire M.K., Gonzales N.R., Lu S., Chitsaz F., Geer L.Y., Geer R.C., He J., Gwadz M., Hurwitz D.I. (2014). CDD. NCBI’s conserved domain database. Nucleic Acids Res..

[50] Marchler-Bauer A., Bo Y., Han L., He J., Lanczycki C.J., Lu S., Chitsaz F., Derbyshire M.K., Geer R.C., Gonzales N.R. (2016). CDD/SPARCLE. functional classification of proteins via subfamily domain architectures. Nucleic Acids Res..

[51] Niu R., Zhou Y., Zhang Y., Mou R., Tang Z., Wang Z., Zhou G., Guo S., Yuan M., Xu G. (2020). uORFlight. a vehicle toward uORF-mediated translational regulation mechanisms in eukaryotes. Database.

[52] Lescot M., Déhais P., Thijs G., Marchal K., Moreau Y., Van de Peer Y., Rouzé P., Rombauts S. (2002). PlantCARE, a database of plant cis-acting regulatory elements and a portal to tools for in silico analysis of promoter sequences. Nucleic Acids Res..

[53] Jin J., Tian F., Yang D.-C., Meng Y.-Q., Kong L., Luo J., Gao G. (2016). PlantTFDB 4.0. toward a central hub for transcription factors and regulatory interactions in plants. Nucleic Acids Res..

[54] Shannon P. (2003). Cytoscape. A Software Environment for Integrated Models of Biomolecular Interaction Networks. Genome Res..

[55] Zhang R., Zhou L., Li Y., Ma H., Li Y., Ma Y., Lv R., Yang J., Wang W., Alifu A. (2022). Rapid Identification of Pollen- and Anther-Specific Genes in Response to High-Temperature Stress Based on Transcriptome Profiling Analysis in Cotton. Int. J. Mol. Sci..

[56] Nurimanguli A.I., Wu Y.L., Pan Z.Y., An Q.S., Shui G.L., Shao P.X., Yang D.Y., Lin H.R., Tang B.H., Xin W.E. Cotton ethylene response factor GhERF91 involved in the defense against Verticillium dahliae. J. Integr. Agric..

[57] Zhu T., Liang C., Meng Z., Sun G., Meng Z., Guo S., Zhang R. (2017). CottonFGD: An integrated functional genomics database for cotton. BMC Plant Biol..

