# Supplementary figures and images for: Genome-Wide Identification and Analysis of the WNK Kinase Gene Family in Upland Cotton
Source: Plants (Basel). 2023 Nov 30;12(23):4036. doi: 10.3390/plants12234036 (PMC10708218; doi:10.3390/plants12234036)

A

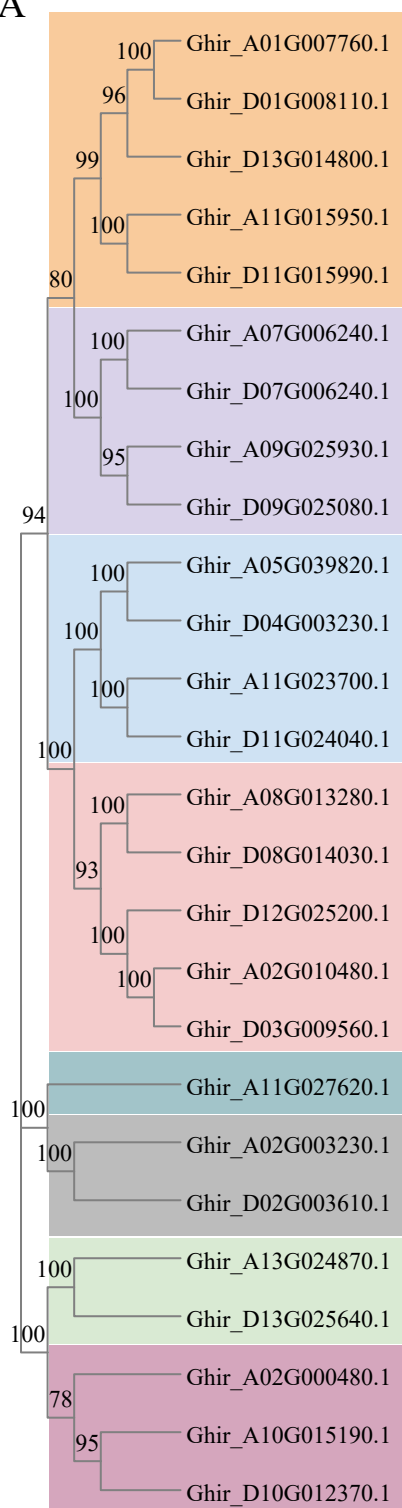

B

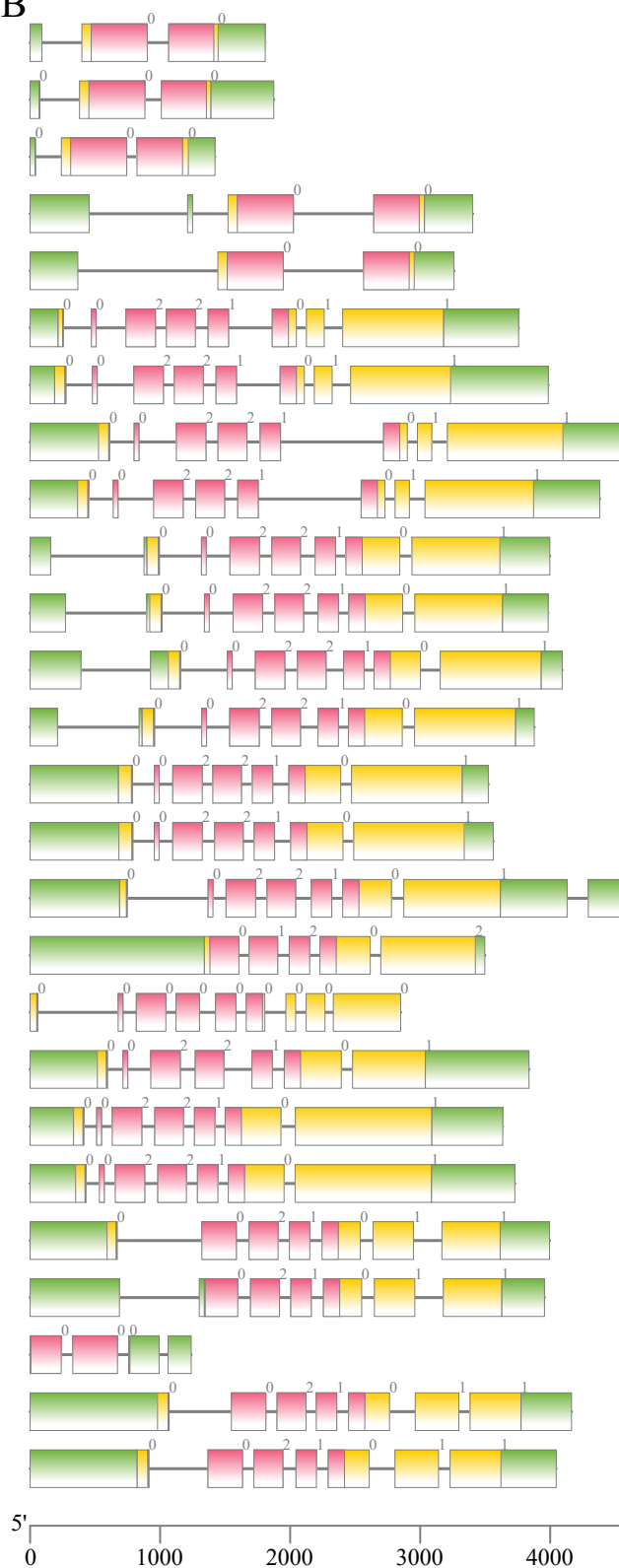

C

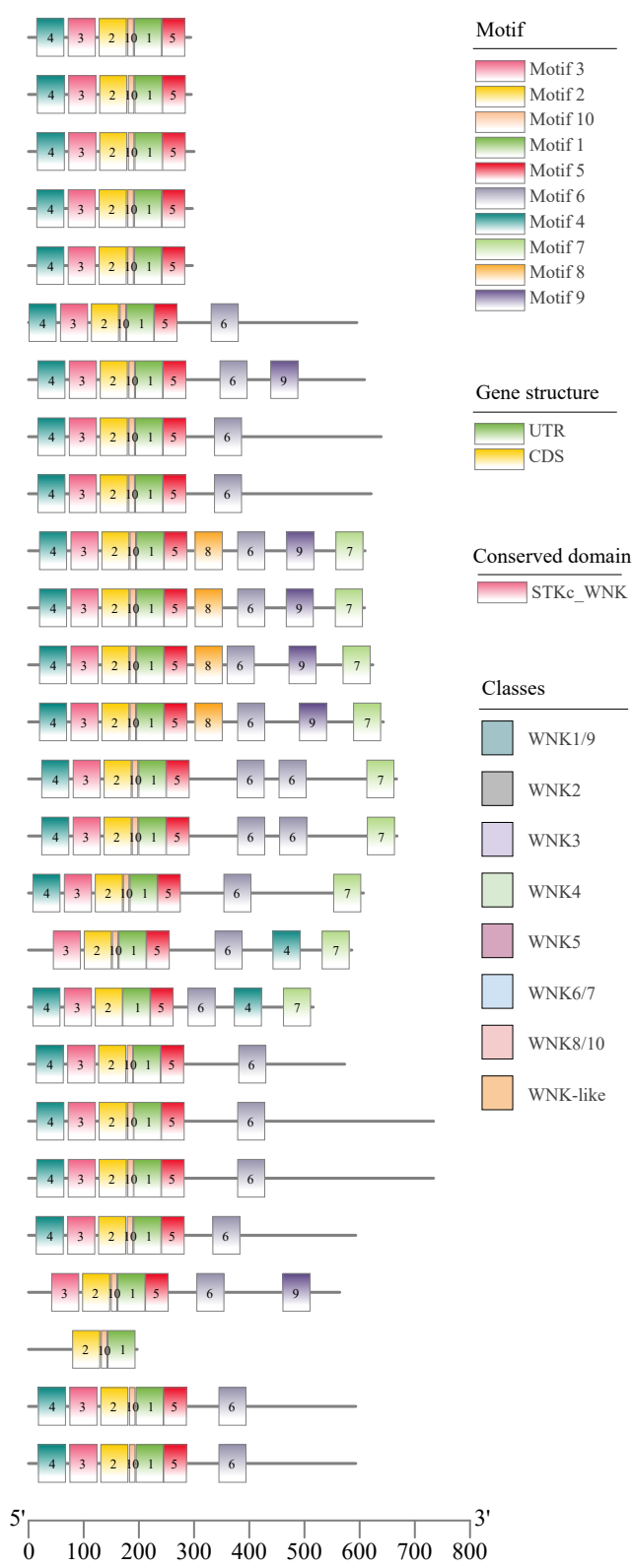

Supplement: Supplementary file 1 [file plants-12-04036-s001.zip › Supplementary Files/fig-2.pdf]

A

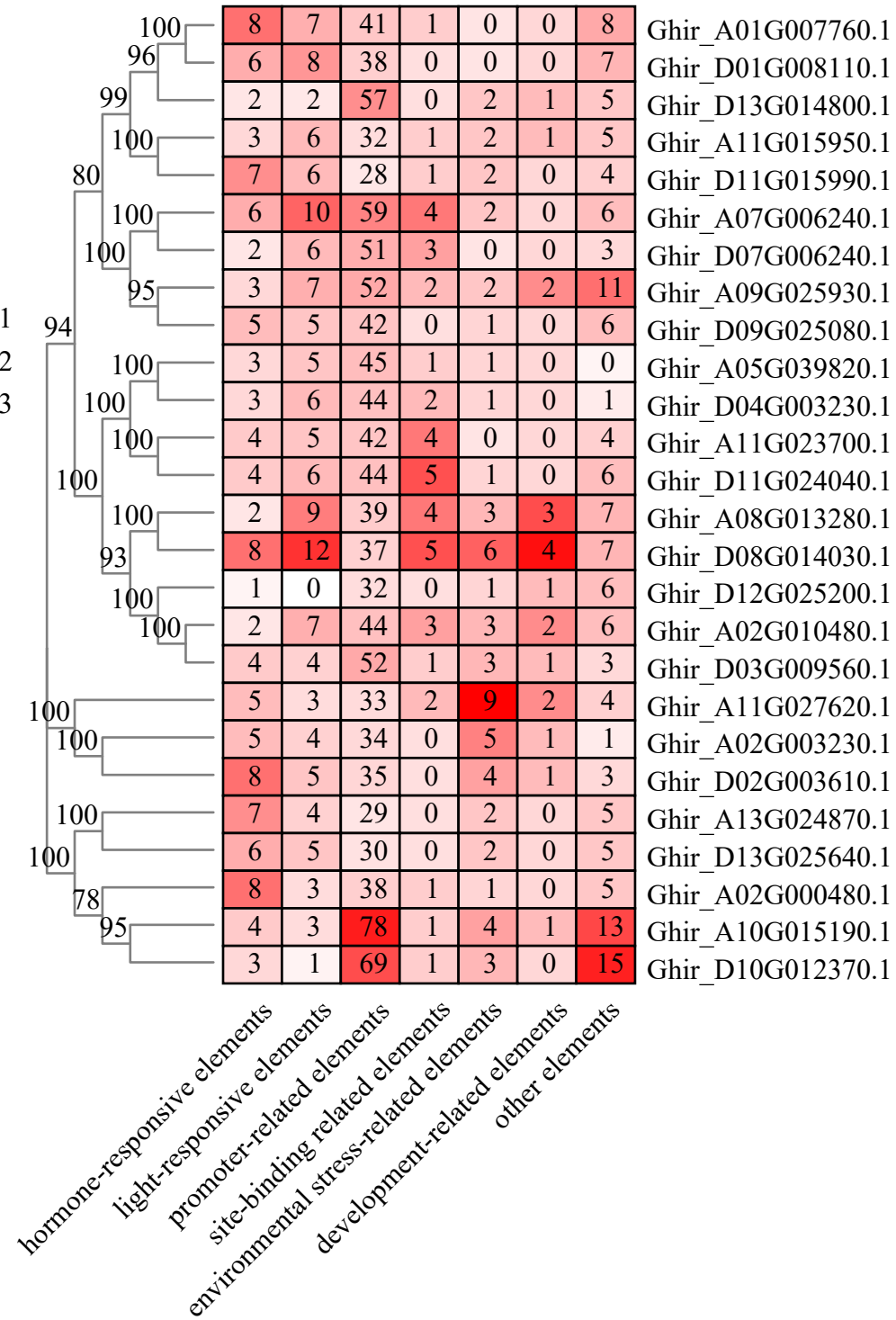

B

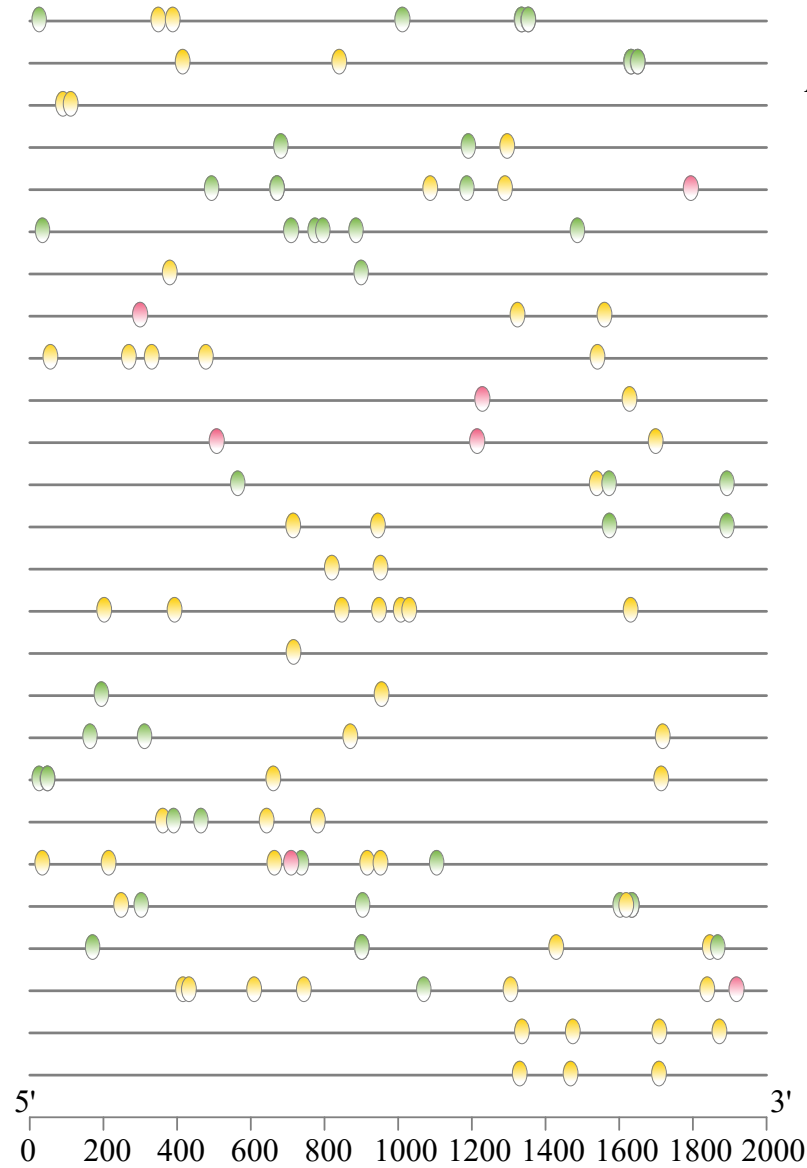

C

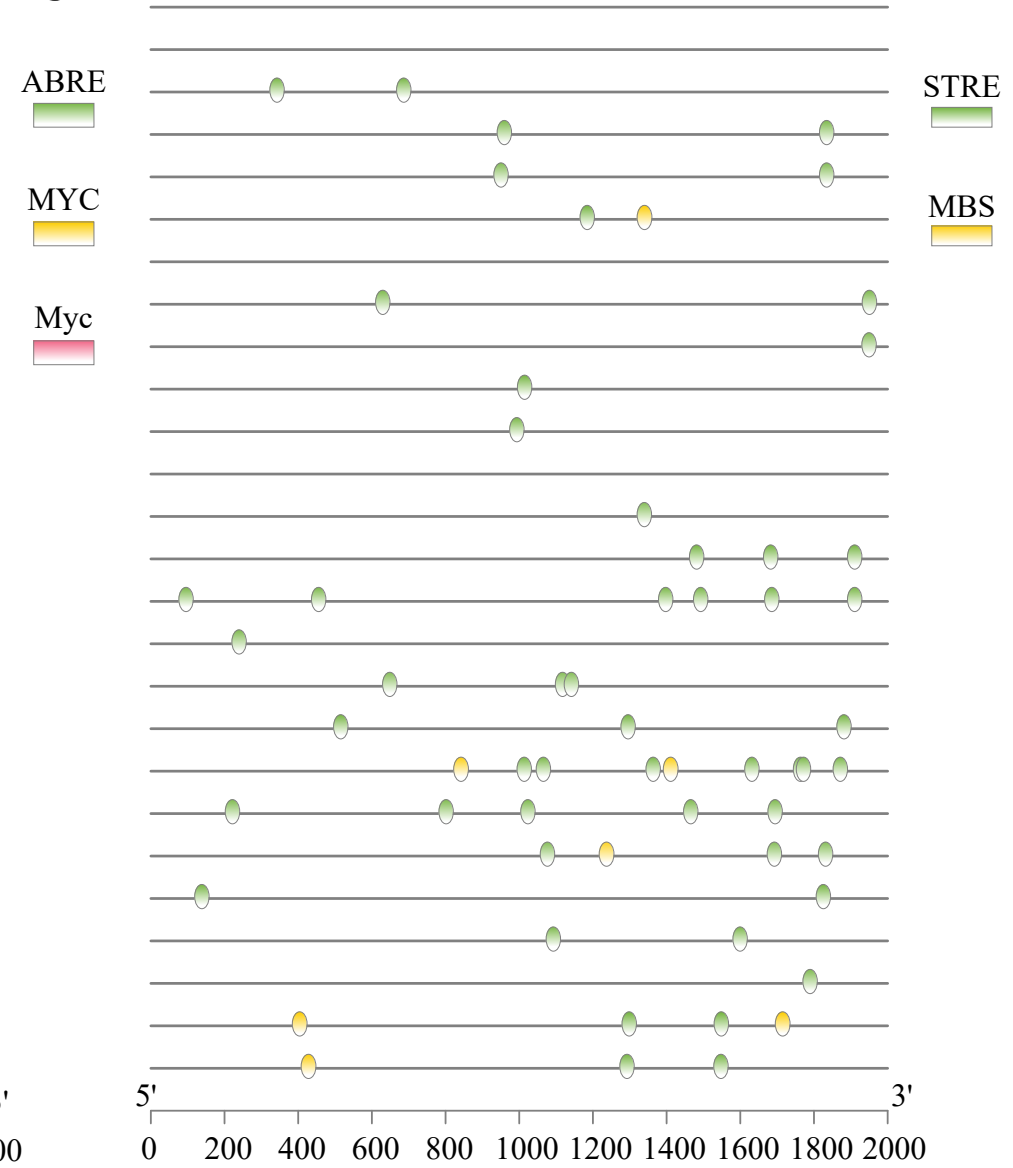

Supplement: Supplementary file 1 [file plants-12-04036-s001.zip › Supplementary Files/fig-3.pdf]
